# Differential expression of Lp-PLA2 in obesity and type 2 diabetes and the influence of lipids

**DOI:** 10.1007/s00125-018-4558-6

**Published:** 2018-02-09

**Authors:** Laura Jackisch, Warunee Kumsaiyai, Jonathan D. Moore, Nasser Al-Daghri, Ioannis Kyrou, Thomas M. Barber, Harpal Randeva, Sudhesh Kumar, Gyanendra Tripathi, Philip G. McTernan

**Affiliations:** 1 Division of Biomedical Sciences, Warwick Medical School, University of Warwick, Coventry, UK; 2 Department of Medical Technology, Chiang Mai University, Chiang Mai, Thailand; 3 Warwick Systems Biology Centre, University of Warwick, Coventry, UK; 4 Biomarkers Research Program, Biochemistry Department, King Saud University, Riyadh, Saudi Arabia; 5 Prince Mutaib Chair for Biomarkers of Osteoporosis, Biochemistry Department, King Saud University, Riyadh, Saudi Arabia; 6 Aston Medical Research Institute, Aston Medical School, Aston University, Birmingham, UK; 7 Human Metabolism Research Unit, Warwickshire Institute for the Study of Diabetes, University Hospitals Coventry and Warwickshire NHS Trust, Coventry, UK; 8 Department of Biomedical Sciences, University of Westminster, 115 New Cavendish Street, London, W1W 6UW UK; 9 College of Science and Technology, Department of Biosciences, Nottingham Trent University, Clifton, Nottingham, NG1 8NS UK

**Keywords:** Adipose tissue, Inflammation, Lipids, Lipoprotein-associated phospholipase A2, Low density lipoprotein, Obesity, Oxidised low density lipoprotein, Type 2 diabetes

## Abstract

**Aims/hypothesis:**

Lipoprotein-associated phospholipase A2 (Lp-PLA2) is a circulatory macrophage-derived factor that increases with obesity and leads to a higher risk of cardiovascular disease (CVD). Despite this, its role in adipose tissue and the adipocyte is unknown. Therefore, the aims of this study were to clarify the expression of Lp-PLA2 in relation to different adipose tissue depots and type 2 diabetes, and ascertain whether markers of obesity and type 2 diabetes correlate with circulating Lp-PLA2. A final aim was to evaluate the effect of cholesterol on cellular Lp-PLA2 in an in vitro adipocyte model.

**Methods:**

Analysis of anthropometric and biochemical variables from a cohort of lean (age 44.4 ± 6.2 years; BMI 22.15 ± 1.8 kg/m^2^, *n* = 23), overweight (age 45.4 ± 12.3 years; BMI 26.99 ± 1.5 kg/m^2^, *n* = 24), obese (age 49.0 ± 9.1 years; BMI 33.74 ± 3.3 kg/m^2^, *n* = 32) and type 2 diabetic women (age 53.0 ± 6.13 years; BMI 35.08 ± 8.6 kg/m^2^, *n* = 35), as part of an ethically approved study. Gene and protein expression of PLA2 and its isoforms were assessed in adipose tissue samples, with serum analysis undertaken to assess circulating Lp-PLA2 and its association with cardiometabolic risk markers. A human adipocyte cell model, Chub-S7, was used to address the intracellular change in Lp-PLA2 in adipocytes.

**Results:**

Lp-PLA2 and calcium-independent PLA2 (iPLA2) isoforms were altered by adiposity, as shown by microarray analysis (*p* < 0.05). Type 2 diabetes status was also observed to significantly alter gene and protein levels of Lp-PLA2 in abdominal subcutaneous (AbdSc) (*p* < 0.01), but not omental, adipose tissue. Furthermore, multivariate stepwise regression analysis of circulating Lp-PLA2 and metabolic markers revealed that the greatest predictor of Lp-PLA2 in non-diabetic individuals was LDL-cholesterol (*p* = 0.004). Additionally, in people with type 2 diabetes, oxidised LDL (oxLDL), triacylglycerols and HDL-cholesterol appeared important predictors, accounting for 59.7% of the variance (*p* < 0.001). Subsequent in vitro studies determined human adipocytes to be a source of Lp-PLA2, as confirmed by mRNA expression, protein levels and immunochemistry. Further in vitro experiments revealed that treatment with LDL-cholesterol or oxLDL resulted in significant upregulation of Lp-PLA2, while inhibition of Lp-PLA2 reduced oxLDL production by 19.8% (*p* < 0.05).

**Conclusions/interpretation:**

Our study suggests adipose tissue and adipocytes are active sources of Lp-PLA2, with differential regulation by fat depot and metabolic state. Moreover, levels of circulating Lp-PLA2 appear to be influenced by unfavourable lipid profiles in type 2 diabetes, which may occur in part through regulation of LDL-cholesterol and oxLDL metabolism in adipocytes.

**Electronic supplementary material:**

The online version of this article (10.1007/s00125-018-4558-6) contains peer-reviewed but unedited supplementary material, which is available to authorised users.


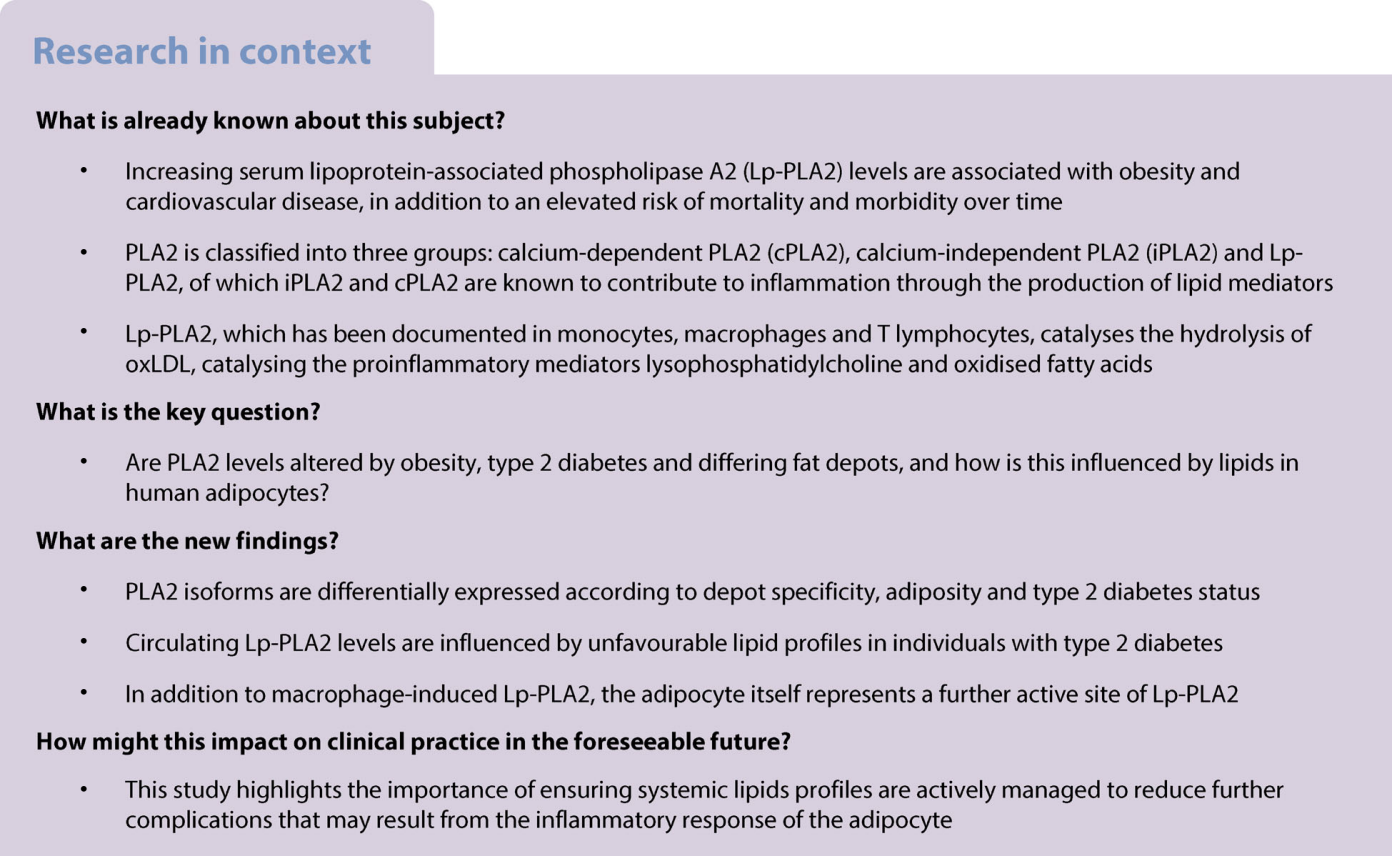


## Introduction

Lipoprotein-associated phospholipase A2 (Lp-PLA2) is a member of the phospholipase A2 superfamily of enzymes, which promote the formation of oxidised LDL (oxLDL), a producer of proinflammatory mediators such as lysophosphatidylcholine and oxidised fatty acids (enhanced in states of metabolic disease) [1, 2]. Prior studies highlight that circulating Lp-PLA2 directly increases arterial inflammation, while cytosolic calcium-dependent PLA2 (cPLA2) and calcium-independent PLA2 (iPLA2) appear to contribute to inflammation via immunological cells [3]. To date, much attention has focused on changes in circulating Lp-PLA2 and disease (arising from epidemiological studies), and has highlighted that Lp-PLA2 is upregulated in conditions of obesity, inflammation and cardiovascular disease (CVD) [4–7].

Macrophage-derived Lp-PLA2 has been shown to promote the instability of vulnerable atherosclerotic plaques, increasing the risk of coronary events; Lp-PLA2 inhibitors have been shown to reduce the frequency of these occurrences [8]. Despite this clear connection with CVD, few studies have explored the impact of circulating Lp-PLA2 in people with type 2 diabetes, which is often considered to precede CVD. The literature suggests that individuals with type 2 diabetes have raised circulating Lp-PLA2 levels, and that these are positively correlated with adiposity and cardiometabolic risk factors [9, 10]. While these studies indicate that increased Lp-PLA2 with adiposity arises as a result of macrophages, the importance of human adipocytes in Lp-PLA2 metabolism remains unclear. This is despite the knowledge that adipocytes possess many overlapping immune cell characteristics, including the production of proinflammatory biomarkers [11, 12].

This study sought to investigate the role of Lp-PLA2 in the adipocyte and the impact of various metabolic states within adipose tissue on Lp-PLA2 expression. Our aims were to: (1) characterise PLA2 gene expression and its related isoforms in adipose tissue; (2) determine the depot-specific expression within adipose tissue and the impact of obesity and type 2 diabetes; (3) define how cardiometabolic risk factors are associated with circulating Lp-PLA2 levels within different metabolic states; and (4) evaluate the molecular impact of LDL-cholesterol and oxLDL on Lp-PLA2 expression within adipocytes.

## Methods

### Participants

Ethical approval was obtained from the Local Research Ethics Committee and all participants gave written and informed consent. For this study, 114 women undergoing elective abdominal surgery were recruited. The cohort consisted of lean (age 44.4 ± 6.2 years; BMI 22.15 ± 1.8 kg/m^2^, *n* = 23), overweight (age 45.4 ± 12.3 years; BMI 26.99 ± 1.5 kg/m^2^, *n* = 24), obese (age 49.0 ± 9.1 years; BMI 33.74 ± 3.3 kg/m^2^, *n* = 32) and women with type 2 diabetes (age 53.0 ± 6.13 years; BMI 35.08 ± 8.6 kg/m^2^, *n* = 35). Detailed medical drug histories were taken and those participants with cancer, thyroid disorders or taking steroids or medication considered to alter inflammatory status, including thiazolidinediones, were excluded.

### Serum and tissue collection

Venous blood samples were taken after an 8–10 h overnight fast. Adipose tissue was obtained by needle biopsy and then flash frozen and/or used for in vitro studies.

### In vivo assessment of biochemical profile

Fasting blood samples were collected from participating volunteers. Lipid profiles and fasting plasma glucose were determined using routine laboratory methods at the University Hospitals Coventry and Warwickshire NHS Trust. In brief, the routine blood tests included glucose and a standard lipidaemic/cholesterol profile (triacylglycerols, HDL-cholesterol and LDL-cholesterol), as noted in Table 1. OxLDL and Lp-PLA2 were measured by ELISA (oxLDL ELISA kit, Mercodia, Uppsala, Sweden; intra-assay %CV = 6.4; inter-assay %CV = 7.4; and Human *PLA2G7*/PAF-AH/Lp-PLA2 Quantikine ELISA, R&D Systems, Abingdon, UK; intra-assay %CV = 6.8, inter-assay %CV = 9.6; respectively). Insulin measurements were performed using a solid-phase enzyme amplified sensitivity multiplex immunoassay (Millipore, Watford, UK), and glucose was measured by a glucose oxidase method (YSL 200 STAT plus, Yellow Springs Instruments, Yellow Springs, OH, USA).

**Table 1:** Selected characteristics of the study participants with and without type 2 diabetes

Characteristic	Non-diabetic			Type 2 diabetic (*n* = 35)
	Lean (*n* = 23)	Overweight (*n* = 24)	Obese (*n* = 32)	
BMI (kg/m^2^)	22.15 ± 1.8	26.99 ± 1.5^***^	33.74 ± 3.2^***^	35.08 ± 8.6^***^
Glucose (mmol/l)	4.61 ± 0.1	4.84 ± 0.1	5.12 ± 0.2^**^	8.56 ± 0.4^***†††^
HOMA-IR	0.89 ± 0.1	1.12 ± 0.1	2.64 ± 0.3^***^	6.21 ± 0.8^***†††^
Cholesterol (mmol/l)	4.55 ± 0.2	4.96 ± 0.2	5.24 ± 0.2^*^	4.97 ± 0.2
Triacylglycerol (mmol/l)	0.76 ± 0.1	0.91 ± 0.1	1.52 ± 0.2^***^	1.44 ± 0.1^***^
LDL-cholesterol (mmol/l)	2.32 ± 0.2	2.73 ± 0.2	3.07 ± 0.1^**^	3.24 ± 0.2^**^
HDL-cholesterol (mmol/l)	1.88 ± 0.1	1.81 ± 0.1	1.45 ± 0.1^**^	1.24 ± 0.1^***†^
LDL-cholesterol /HDL-cholesterol	1.31 ± 0.5	1.66 ± 0.2	2.39 ± 0.2^***^	2.87 ± 0.2^***†^
Insulin (pmol/l)	28.95 ± 8.0	33.24 ± 7.6	69.45 ± 7.6^***^	91.55 ± 7.8^***†^
Endotoxin (EU/ml)	2.12 ± 0.2	2.91 ± 0.3^*^	4.39 ± 0.4^***^	6.95 ± 0.3^***†††^
OxLDL (U/l)	39.17 ± 1.5	41.08 ± 2.5	51.87 ± 3.1^***^	66.88 ± 4.4^***††^
Lp-PLA2 (pmol/l)	2.39 ± 0.14	2.81 ± 0.21	2.98 ± 0.19^*^	2.94 ± 0.16^*^

Data are means ± SEM, except for BMI which is mean ± SD

Unpaired *t* test was used to compare means

*p < 0.05, **p < 0.01 and ***p < 0.001 for lean vs overweight, obese and type 2 diabetic

†*p* < 0.05, ††*p* < 0.01 and †††*p* < 0.001 for obese vs type 2 diabetic

### Analysis of circulating endotoxins

Serum endotoxin was analysed using the QCL-1000 LAL endpoint assay (Lonza, Allendale, NJ, USA). The assay, and the values given by the manufacturer for intra-assay %CV (3.9 ± 0.46%) and inter-assay %CV (9.6 ± 0.75%), have been validated in our laboratory, as detailed previously [13, 14].

### Isolation of pre-adipocytes, stromal vascular fraction and mature adipocytes

Abdominal subcutaneous (AbdSc) adipose tissue was digested as previously described to isolate stromal vascular fraction (SVF), pre-adipocytes and mature adipocytes [15]. In short, adipose tissue was incubated with collagenase for 30 min, the digest was then filtered through a cotton mesh and centrifuged. Differential centrifugation resulted in floating mature adipocytes and pellets of SVF. The pre-adipocytes were cultured, while RNA was extracted directly from SVF and mature adipocytes.

### Protein determination and western blot analysis

A subgroup of paired human AbdSc and omental adipose tissue biopsies from participants who were lean (age 43.6 ± 6.2 years; BMI 22.5 ± 2.2 kg/m^2^; *n* = 9), overweight (age 47.5 ± 11.5 years; BMI 27.4 ± 1.5 kg/m^2^; *n* = 10) or obese (age 48.1 ± 8.5 years; BMI 34.0 ± 2.9 kg/m^2^; *n* = 5) was used for protein analysis. The adipose tissue was homogenised in Phosphosafe extraction buffer (Novagen, Merck, Darmstadt, Germany) and cultured adipocytes were harvested in RIPA buffer (Cell Signaling, Denver, MA, USA) with a cocktail of protease inhibitors, to extract total protein. Protein concentrations were measured using the Bio-Rad Detergent Compatible protein assay kit (Bio-Rad, San Diego, CA, USA) [16]. Western blotting was performed as described elsewhere [17], and protein levels of cPLA2 (1:100, Cell Signaling), iPLA2 (1:500, Sigma, Poole, UK) and Lp-PLA2 (1:200, R&D Systems) were assessed with rabbit and goat monoclonal antibodies.

### RNA extraction and quantitative PCR

RNA was extracted from samples using an RNeasy lipid tissue kit (Qiagen, Manchester, UK) according to the manufacturer’s instructions, followed by a DNase digestion step. cDNA was synthesised using reverse transcription reagents (Bioline, London, UK). Quantitative (q)PCR was performed with TaqMan probes (18S, Hs03003631_g1; *PLA2G7*, Hs00173726_m1; *PLA2G4*, Hs00233352_m1; *PLA2G6*, Hs00185926_m1; *CD68*, Hs02836816_g1; *CD206* [also known as *MRC1*], Hs00267207_m1; *HLA-DRA*, Hs00219575_m1; *CIITA*, Hs00172106_m1; *EMR1* [also known as *ADGRE1*], Hs00173562_m1; Applied Biosystems, Warrington, UK). Transcript abundance was measured with an Applied Biosystems 7500 Real-Time PCR System with TaqMan universal PCR master mix. All reactions were multiplexed with the housekeeping gene 18S, to normalise qPCR data.

### Immunohistochemistry

Adipose and placenta tissue samples were incubated with primary polyclonal Lp-PLA2 antibody (R&D Systems) in a dilution of 1:100. Sections were developed using peroxidase substrate kit VIP (Vector Laboratories, Peterborough, UK) for Lp-PLA2. To demonstrate specific binding, the primary antibody was omitted for negative control for Lp-PLA2 independently.

### Microarray analysis

RNA from the adipose tissue samples was used for gene expression analysis with the Human Genome U133A plus 2.0 DNA microarrays (Affymetrix, Santa Clara, CA, USA). Preparation of cRNA and hybridisation to DNA microarrays were performed according to standard Affymetrix protocols, as previously described [18, 19]. PLA2 mRNA expression was investigated using the 219064_AT probe set.

### Cell cultures

AbdSc pre-adipocytes were grown to confluence in DMEM/F-12 containing 10% (vol./vol.) fetal bovine serum, 1% (vol./vol.) penicillin/streptomycin and transferrin (62.5 pmol/l) at 37°C, 5% CO_2_ incubation. For cell differentiation, AbdSc pre-adipocytes were maintained in PromoCell pre-adipocyte differentiation media (PromoCell, Heidelberg, Germany) for 48 h. Subsequently, the cells were maintained in the PromoCell adipocyte nutrition media (PromoCell, Heidelberg, Germany) for 14 days, with the medium changed every 2 days. The differentiated adipocytes were then given a 24 h wash out in DMEM/F12 supplemented with 0.5% (wt/vol.) BSA. Chub-S7 cells, a human AbdSc pre-adipocyte cell line, were grown under the same conditions [20].

The differentiated AbdSc Chub-S7 cells (*n* = 6) were treated with LDL-cholesterol (67 pmol/l) ± 20 μmol/l of the Lp-PLA2 inhibitor (Darapladib, Cayman Chemical, Ann Arbor, Michigan, USA) or oxLDL (43 pmol/l; Kalen Biomedical, Germantown, MD, USA) for 3, 6, 24 and 48 h. PBS containing 0.34 mmol/l EDTA was used as the control. Experiments were conducted with six technical replicates per treatment.

### Statistical analysis

For microarray data analysis, one-way ANOVA was performed for each selected PLA2 gene. Significance of mRNA expression and protein levels in different adiposity, fat depot and type 2 diabetes status data were analysed with paired *t* tests. For the cell culture, treatments were compared using two-way ANOVA. All quantitative variables are shown as the mean ± SEM, unless otherwise stated.

Determination of correlations in gene expression analysis was performed using Pearson’s correlation coefficient. Spearman’s rank correlation coefficient was used to determine correlations for serum Lp-PLA2 and metabolic markers because of the non-parametric distribution. Furthermore, multivariate stepwise regression was applied to calculate predictors of systemic Lp-PLA2 and Bonferroni correction was used to adjust the *p* value to 0.0045. Power analyses to determine sample size were carried out using G^*^Power version 3.1.9.2 (G*power, Düsseldorf, Germany). Analyses and graphing were performed using SPSS version 18.0 for Windows (SPSS, Chicago, Illinois, USA) and GraphPad Prism version 7.01 (GraphPad, La Jolla, CA, USA), respectively. Levels of statistical significance were set at *p < 0.05, **p < 0.01 and ***p < 0.001, unless otherwise stated.

## Results

### PLA2 microarray expression profile in adipose tissue

**Fig. 1:**
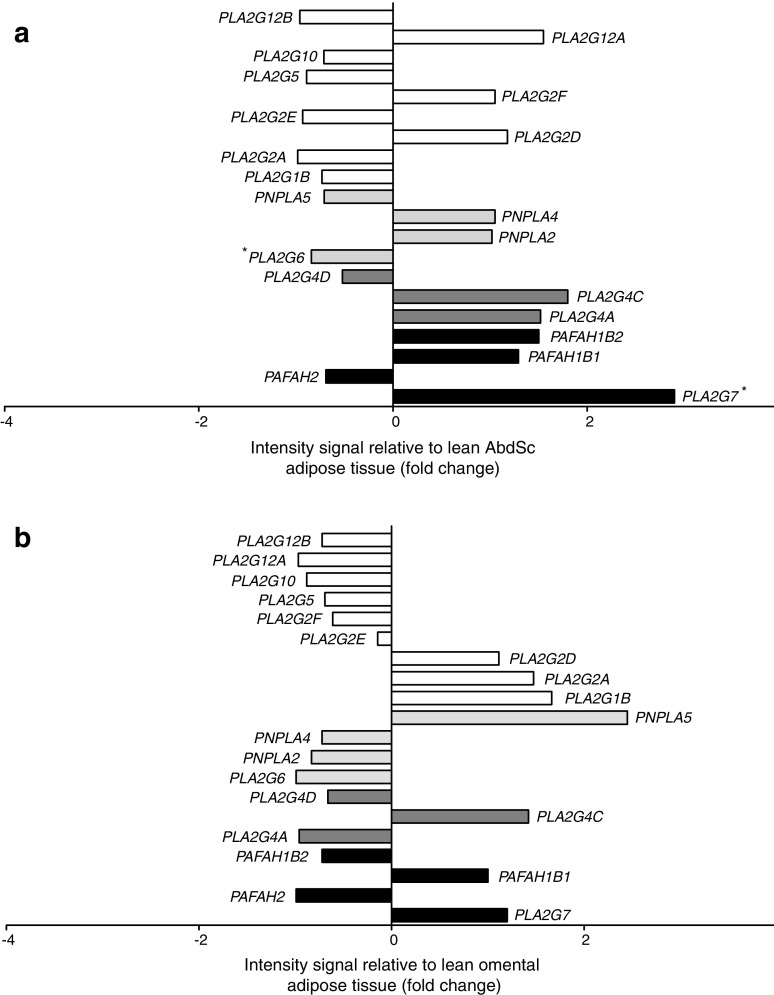
Microarray data analysis of the PLA2 gene family. The data are represented as a ratio between intensity signal from lean and obese (**a**) AbdSc adipose tissue and (**b**) omental adipose tissue. Statistical analysis was performed using one-way ANOVA. White bars, secretory PLA2; light grey bars, cPLA2; dark grey bars, iPLA2; black bars, platelet-activating factor acetylhydrolase. *p < 0.05

A microarray approach containing 20 probes was used to obtain a comprehensive picture of genes differentially expressed in AbdSc and omental adipose tissue from lean and obese participants. The probes corresponded to genes encoding for four isoenzymes in the platelet-activating factor acetylhydrolase family (*PLA2G7*, *PAFAH2*, *PAFAH1B1* and *PAFAH1B2*), three isoenzymes in the cPLA2 family (*PLA2G4A*, *PLA2G4C* and *PLA2G4D*), four isoenzymes in the iPLA2 family (*PLA2G6*, *PNPLA2*, *PNPLA4* and *PNPLA5*) and nine isoenzymes in the secretory PLA2 family (*PLA2G1B*, *PLA2G2A*, *PLA2G2D*, *PLA2G2E*, *PLA2G2F*, *PLA2G5*, *PLA2G10*, *PLA2G12A* and *PLA2G12B*). Increased expression (*p* < 0.05) was noted for *PLA2G7* (encoding Lp-PLA2), while *PLA2G6* (encoding iPLA2) showed downregulation (*p* < 0.05) in AbdSc adipose tissue taken from obese individuals compared with lean individuals (Fig. 1a). Our raw array data are available online at Open Science Network (https://osf.io/s6rw3/).

### PLA2 family mRNA and protein levels in adipose tissue

To further characterise PLA2 in relation to adiposity, we investigated *PLA2G7*, *PLA2G6* and *PLA2G4* (encoding cPLA2) expression in subcutaneous and omental adipose tissue sampled from lean, overweight and obese individuals.

**Fig. 2:**
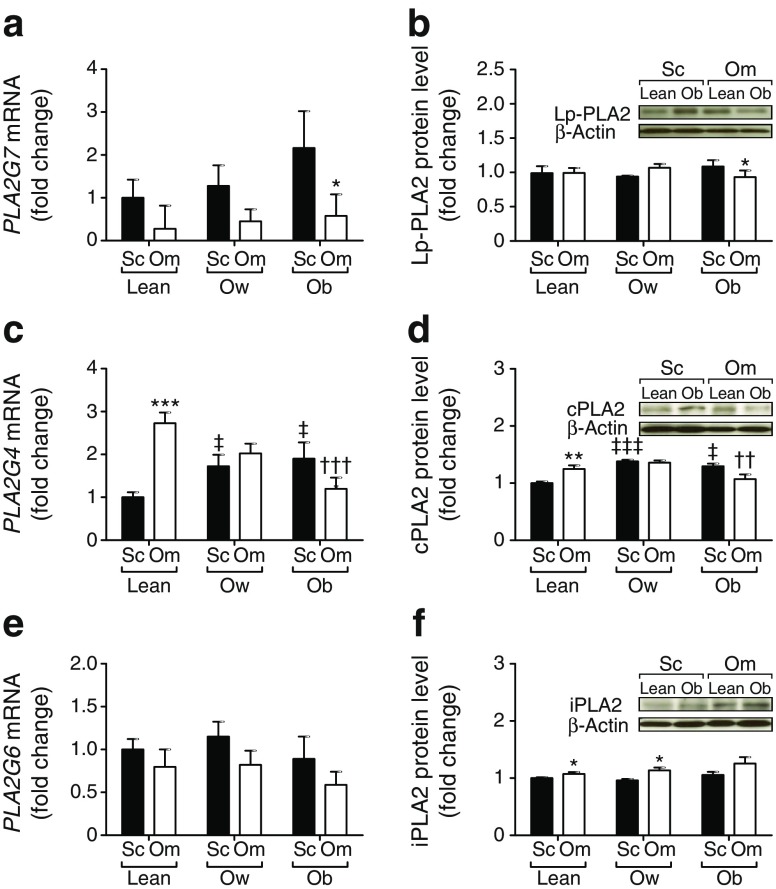
mRNA and protein expression of PLA2 in AbdSc adipose tissue and omental adipose tissue from lean (*n* = 9), overweight (*n* = 10) and obese (*n* = 5) non-diabetic individuals. (**a**) *PLA2G7* mRNA expression. (**b**) Lp-PLA2 protein levels. (**c**) *PLA2G4* mRNA expression. (**d**) cPLA2 protein levels. (**e**) *PLA2G6* mRNA expression. (**f**) iPLA2 protein levels. All qPCR and western blot results were standardised to lean AbdSc adipose tissue. Statistical analysis was performed using paired *t* test. *p < 0.05, **p < 0.01 and ***p < 0.001 for AbdSc vs omental adipose tissue; ††*p* < 0.01 and †††*p* < 0.001 vs lean omental adipose tissue; and ‡*p* < 0.05 and ‡‡‡*p* < 0.05 vs lean AbdSc adipose tissue. Ob, obese; Om, omental adipose tissue; Ow, overweight; Sc, subcutaneous

The level of *PLA2G7* mRNA was significantly higher in AbdSc than omental adipose tissue in obese participants (*p* < 0.05); no difference was observed between the other groups (Fig. 2a). *PLA2G7* gene expression was raised in adipose tissue samples from overweight or obese individuals compared with lean individuals, but the difference was not significant (Fig. 2a). Analysis of Lp-PLA2 protein indicated that increasing adiposity alone did not increase Lp-PLA2 levels in adipose tissue (Fig. 2b).

Analysis of *PLA2G4* gene expression demonstrated that *PLA2G4* was expressed preferentially in OM adipose tissue depots of lean individuals rather than AbdSc adipose tissue (*p* < 0.001; Fig. 2c). Overweight and obese individuals had higher levels of *PLA2G4* gene expression in the AbdSc adipose tissue depot compared with their lean counterparts (*p* < 0.05). Interestingly, in the omental adipose tissue depot, *PLA2G4* mRNA expression was significantly decreased in the obese participants (*p* < 0.001; Fig. 2c). Subsequent cPLA2 protein analysis confirmed the gene expression findings for AbdSc and omental adipose tissue (Fig. 2d).

Next, we investigated *PLA2G6* gene expression between paired AbdSc and omental adipose tissue and no effect of adiposity was observed (Fig. 2e). Protein analysis, however, revealed that levels of iPLA2 were higher in omental than AbdSc adipose tissue in lean and overweight participants (*p* < 0.05; Fig. 2f). Taken together, these results indicate that depot specificity and differing levels of adiposity affect PLA2 expression in adipose tissue.

### Influence of type 2 diabetes status on Lp-PLA2

**Fig. 3:**
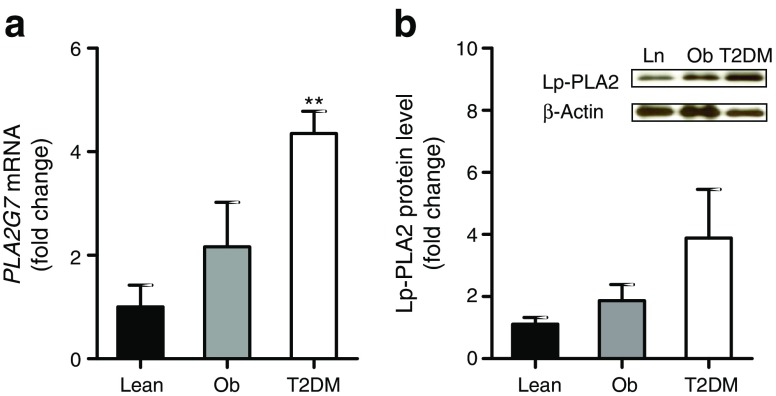
Influence of type 2 diabetes status on mRNA expression and protein levels of Lp-PLA2. (**a**) *PLA2G7* mRNA expression and (**b**) Lp-PLA2 protein expression in AbdSc adipose tissue in lean, obese and type 2 diabetic individuals. Statistical analysis was performed using paired *t* tests. **p < 0.01 vs lean AbdSc adipose tissue. Ln, lean; Ob, obese; T2DM, type 2 diabetes

Given that PLA2 levels were markedly altered by adiposity, we investigated the influence of type 2 diabetes status. Analysis of *PLA2G7* gene expression in AbdSc adipose tissue depots from lean, obese and type 2 diabetic individuals showed a significant increase in diabetic adipose tissue compared with lean adipose tissue (*p* < 0.01; Fig. 3a). Consistent with these findings, protein analysis demonstrated increased levels of Lp-PLA2 in individuals with type 2 diabetes; however, this increase was more modest and did not reach statistical significance (Fig. 3b). There were no significant differences in AbdSc adipose tissue mRNA and protein levels of cPLA2 and iPLA2 between the non-diabetic and type 2 diabetic groups (data not shown). Thus, type 2 diabetes status appeared to be associated with an upregulation of Lp-PLA2 in adipose tissue.

### Analysis of Lp-PLA2 and macrophage markers in adipocytes and adipose tissue

**Fig. 4:**
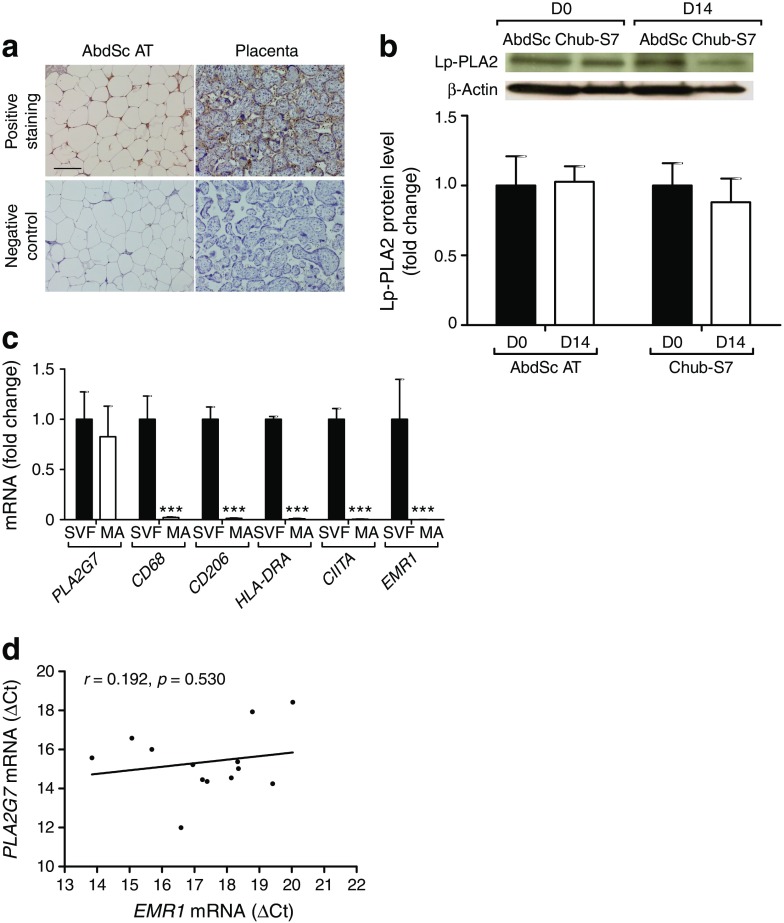
Analysis of Lp-PLA2 and macrophage markers in adipocytes and adipose tissue. (**a**) Representative images of immunohistochemical staining in human AbdSc adipose tissue and placenta, showing adipocytes expressing Lp-PLA2. (**b**) Lp-PLA2 protein levels in human primary culture and a human adipocyte cell line (Chub-S7) on day 0 and day 14 of differentiation. (**c**) Gene expression of *PLA2G7* (Lp-PLA2) and macrophage markers in SVF and mature adipocytes. (**d**) Correlation between mRNA levels of *PLA2G7* and *EMR1*, encoding the macrophage-specific marker EMR1, in AbdSc adipose tissue from type 2 diabetic individuals. The solid line represents linear regression. Statistical analysis was performed using two-way ANOVA (**d**) and paired *t* test (**b**, **c**). Scale bar, 100 μm. ***p < 0.001. AT, adipose tissue; D, day; MA, mature adipocyte

In view of the changes of Lp-PLA2 in adipose tissue, our study further sought to investigate the source of Lp-PLA2 in adipose tissue. Using immunohistochemical staining, we observed the expression of Lp-PLA2, as denoted by the brown staining observed around each cell, in mature adipocytes from lean non-diabetic individuals (Fig. 4a). Placental tissue was used as a positive control for Lp-PLA2 staining, with positive brown staining also shown, in addition to negative staining noted in both tissue sections (Fig. 4a).

Consistent with these findings, protein analysis demonstrated the presence of Lp-PLA2 in a pure human adipocyte cell line, Chub-S7, pre- and post-differentiation (Fig. 4b). Analysis of Lp-PLA2 content in cultured primary human adipocytes from AbdSc adipose tissue also revealed the presence of Lp-PLA2 in both pre-adipocytes and mature adipocytes.

Given that macrophages are known to express Lp-PLA2 in adipose tissue, we determined the expression of macrophage markers in mature adipocyte samples. No differences were observed in *PLA2G7* mRNA expression between mature adipocytes and SVF isolated from adipose tissue after collagenase digestion (Fig. 4c). Mature adipocytes had reduced levels of the macrophage markers *CD68*, *CD206*, *HLA-DRA*, *CIITA* and *EMR1* in comparison with SVF (Fig. 4c). Additionally, no significant correlations were identified between the gene expression of these macrophage markers and *PLA2G7* (see electronic supplementary material [ESM] Fig. 1a-e). Similarly, correlation analysis of *EMR1* and *PLA2G7* mRNA expression in AbdSc adipose tissue showed no significant correlation (*r* = 0.192, *p* = 0.530; Fig. 4d). The results presented here clearly demonstrate the expression of Lp-PLA2 by adipocytes from human adipose tissue.

### Comparison of anthropometric and biochemical analytes in subcohorts

The association of Lp-PLA2 with selected metabolic markers was investigated to further understand the influence of metabolic states on circulating Lp-PLA2.

Table 1 shows baseline characteristics from fasted participants in four subcohorts. Obese participants and those with type 2 diabetes status had significantly higher levels of Lp-PLA2 compared with the lean group (*p* < 0.05); this association was attenuated in the overweight group. Compared with the lean study group, the obese and type 2 diabetic women also had statistically different levels of all other metabolic markers, apart from cholesterol in the obese group. These associations were not seen in the overweight participants, except for endotoxin levels, which were significantly increased (*p* < 0.05).

**Fig. 5:**
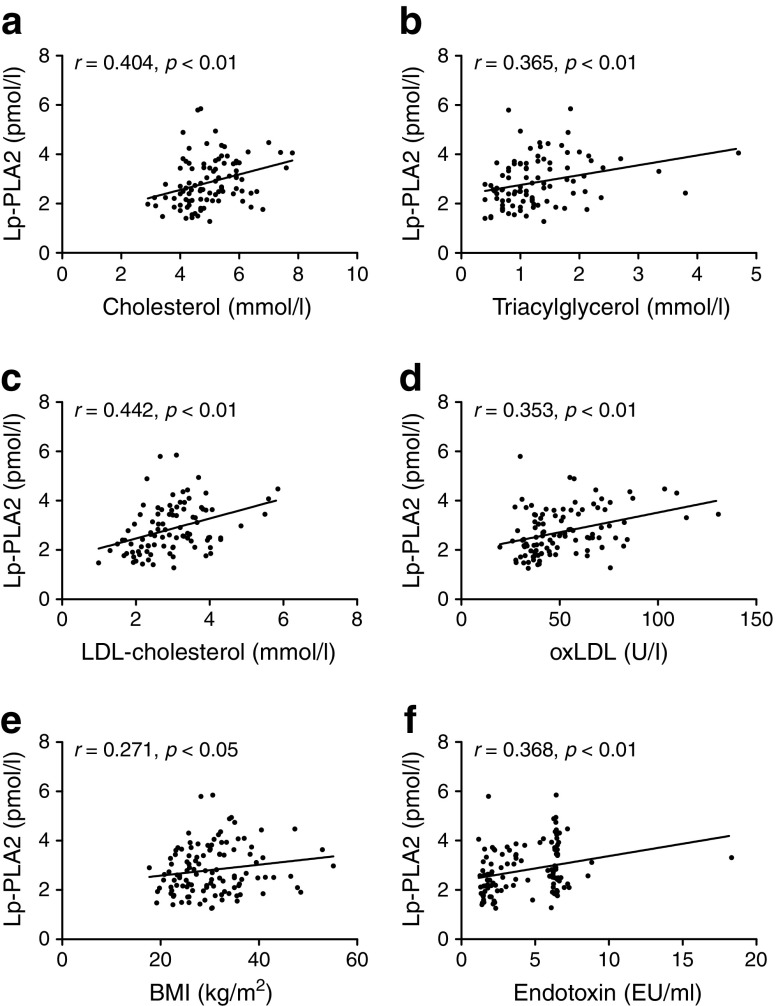
Correlations between Lp-PLA2 (pmol/l) and: (**a**) cholesterol (mmol/l), (**b**) triacylglycerol (mmol/l), (**c**) LDL-cholesterol (mmol/l), (**d**) oxLDL (U/l), (**e**) BMI (kg/m^2^) and (**f**) endotoxin (EU/ml). Correlation analysis was performed using Spearman’s rank correlation analysis, followed by two-way ANOVA

The relationships between circulating Lp-PLA2 and key metabolic markers were determined using linear regression. Analysis of our full cohort revealed that Lp-PLA2 positively correlated with cholesterol, LDL-cholesterol, oxLDL, BMI and endotoxin (Fig. 5). Subsequent subcohort analysis revealed significant positive correlations with metabolic markers, including cholesterol, triacylglycerol, LDL-cholesterol, LDL-cholesterol /HDL-cholesterol, endotoxin and oxLDL in non-diabetic individuals (Table 2). Additionally, strong correlations between Lp-PLA2 and HDL-cholesterol, LDL-cholesterol/HDL-cholesterol and oxLDL were maintained in individuals with type 2 diabetes.

**Table 2:** Correlations between serum Lp-PLA2 and selected metabolic markers

Characteristic	Non-diabetic				Type 2 diabetic (*n* = 35)
	Non-diabetic (*n* = 79)	Lean (*n* = 23)	Overweight (*n* = 24)	Obese (*n* = 32)	
BMI	0.271^*^	0.146	0.113	0.138	−0.042
Glucose	0.123	0.306	0.011	0.025	0.236
HOMA-IR	0.249	0.395	0.468	0.215	0.143
Cholesterol	0.404^**^	0.537^*^	0.310	0.315	0.176
Triacylglycerol	0.365^**^	0.459	0.288	0.299	0.146
LDL-cholesterol	0.442^**^	0.607^*^	0.221	0.400^*^	0.306
HDL-cholesterol	−0.281^*^	0.207	0.113	0.420^*^	−0.536^**^
LDL-cholesterol /HDL-cholesterol	0.449^***^	0.596^*^	0.025	0.486^**^	0.588^***^
Insulin	0.304^*^	0.297	0.465	0.250	0.122
Endotoxin	0.368^**^	0.590^**^	0.125	0.326	0.071
OxLDL	0.353^**^	0.460^*^	0.196	0.328	0.657^**^

The values shown are Spearman’s correlation coefficients

*p < 0.05, **p < 0.01 and ***p < 0.001

Thereafter, multivariate stepwise regression analysis was used to identify factors that influence circulating Lp-PLA2 across non-diabetic and diabetic groups. In the whole study population, LDL-cholesterol was the sole determinant of circulating Lp-PLA2, accounting for 9.7% of the variance observed (Table 3). Further analysis of type 2 diabetic individuals revealed that oxLDL, triacylglycerol and HDL-cholesterol account for 59.7% of the variance (*p* < 0.001). In non-diabetic individuals, LDL-cholesterol was a significant predictor (*p* = 0.02), but not after the Bonferroni correction was applied.

**Table 3:** Variables statistically associated with circulating Lp-PLA2 in multivariate stepwise regression analysis

Independent variables	Standard β^a^	Adjusted *R*^2b^	*p* value for *R*^2^
Non-diabetic	–	0.096	0.020
LDL-cholesterol	0.34 ± 0.18	–	–
Type 2 diabetic	–	0.597	1.5×10^−5^
OxLDL	0.77 ± 0.09	–	–
Triacylglycerol	−0.38 ± 0.13	–	–
HDL-cholesterol	−0.30 ± 0.14	–	–
All	–	0.097	0.004
LDL-cholesterol	0.33 ± 0.11	–	–

The following independent variables were considered for the model: BMI, fasting glucose, HOMA-IR, cholesterol, triacylglycerol, LDL-cholesterol, HDL-cholesterol, insulin, endotoxin, oxLDL

^a^Standardised regression β coefficient ± SEM

^b^Adjusted coefficient of determination

*p* values for adjusted *R*^2^ are significant at <0.0045 (Bonferroni adjusted *p* value)

These findings indicate that oxLDL, triacylglycerol and HDL-cholesterol are the most important predictors of Lp-PLA2 in patients with type 2 diabetes; additionally, correlation data show a clear link between lipid profile and Lp-PLA2 across different metabolic states.

### Effect of Lp-PLA2 inhibitor, oxLDL and LDL-cholesterol on Chub-S7 cells

Given that our study revealed an association between Lp-PLA2 and the unfavourable circulating lipid profile in type 2 diabetic individuals, we sought to determine the direct influence of lipid mediators on Lp-PLA2 activity.

**Fig. 6:**
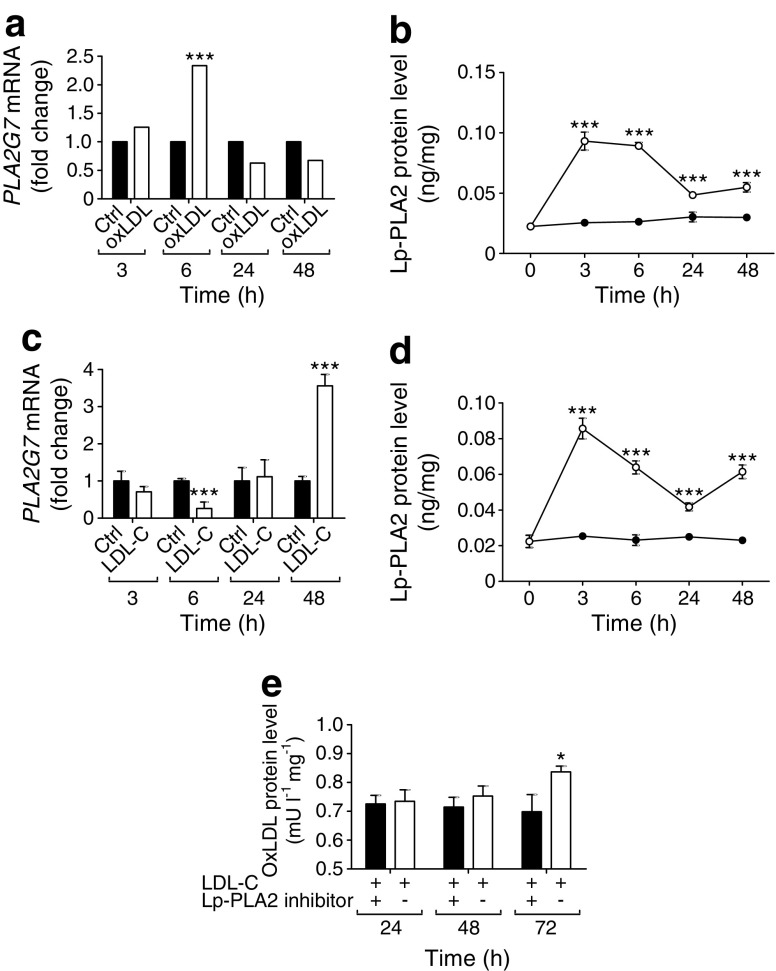
Effects of an Lp-PLA2 inhibitor, oxLDL and LDL-cholesterol on Lp-PLA2 gene expression and protein levels. Differentiated Chub-S7 adipocytes (*n* = 6) were treated for 3, 6, 24 and 48 h with: (**a**) 43 pmol/l oxLDL, *PLA2G7* mRNA was measured; (**b**) 43 pmol/l oxLDL, Lp-PLA2 protein levels were measured (black circles, control; white circles, oxLDL-treated cells); (**c**) 67 pmol/l LDL-cholesterol, *PLA2G7* mRNA was measured; and (**d**) 67 pmol/l LDL-cholesterol, Lp-PLA2 protein levels were measured (black circles, control; white circles, LDL-cholesterol-treated cells). (**e**) Cells were treated with 200 μg protein/ml LDL-cholesterol and with or without 20 μmol/l Lp-PLA2 inhibitor for 24, 48 and 72 h. The oxLDL levels (mU/l) were normalised to individual total protein concentration. Statistical analysis was performed using two-way ANOVA. *p < 0.05 and ***p < 0.001 vs 0 h. Ctrl, control; LDL-C, LDL-cholesterol

Treatment of Chub-S7 cells with oxLDL induced an acute rise in *PLA2G7* mRNA levels that was statistically significant at 6 h post treatment, and which slowly declined up until 48 h (*p* < 0.001; Fig. 6a). Protein levels displayed similar results, with upregulation of Lp-PLA2 at 3 h and 6 h, followed by a steep decline post 6 h, although still significantly higher than the control (*p* < 0.001, Fig. 6b). Treatment with native LDL-cholesterol resulted in increased *PLA2G7* gene expression at 48 h compared with 3 h (*p* < 0.001; Fig. 6c). However, 6 h stimulation with LDL-cholesterol diminishes *PLA2G7* gene expression (*p* < 0.001; Fig. 6c). Interestingly, Lp-PLA2 protein levels increased at every time point post LDL-cholesterol treatment (*p* < 0.001, Fig. 6d).

Further analysis of LDL treatment explored whether adipocytes were converting LDL into oxLDL, and how this conversion was affected by an Lp-PLA2 inhibitor. At 72 h incubation, the oxLDL level had significantly increased by 19.8% compared with 24 h incubation (*p* < 0.05) (Fig. 6e). The increased oxLDL production in medium significantly diminished when the cells were treated with LDL-cholesterol plus Lp-PLA2 inhibitor (Fig. 6e). Thus, these studies indicate a functional requirement for Lp-PLA2 in adipocytes for oxLDL production.

## Discussion

The main purpose of this study was to evaluate the role of the adipocyte as a source of Lp-PLA2, and its capacity to influence oxLDL production as a contributing influence in inflammation within obesity-mediated diabetes. To establish this, we comprehensively evaluated PLA2 isoforms in different states of adiposity and diabetes status, noting the effect of cholesterol on cellular Lp-PLA2 in an in vitro adipocyte model. We report that PLA2 and its isoforms appear to be heavily influenced by weight, metabolic state and circulating lipids. Moreover, Lp-PLA2 is associated with an unfavourable circulating lipid profile, including increased oxLDL and triacylglycerol, which is exacerbated in type 2 diabetes. Furthermore, our in vitro adipocyte studies show that Lp-PLA2 is expressed and functional, being influenced by LDL-cholesterol and oxLDL in a similar fashion to monocyte studies [21].

Obesity contributes to a heightened inflammatory response in adipose tissue [22]; the influence of the PLA2 superfamily in this condition has been unclear to date [23]. Its importance in adipose tissue may arise as cPLA2 is phosphorylated by c-Jun N-terminal kinase (JNK), p38 mitogen-activated protein kinase (MAPK) and p42/44 MAPKs—all known intracellular mediators of inflammation [24]. Similarly, iPLA2 and Lp-PLA2 are also considered pathogenic inflammatory markers in monocytes [25, 26]. Our microarray analysis of PLA2 isoforms in AbdSc adipose tissue found that Lp-PLA2 and iPLA2 levels were altered by adiposity, but this was not mirrored by subsequent gene or protein expression data. However, cPLA2 mRNA and protein levels increased with adiposity. The apparent disparity between our initial microarray results and subsequent mRNA and protein analysis may have reflected the limited number of participants available for microarray analysis. Furthermore, analysis of PLA2 isoforms in adipose tissue from a male cohort (given their higher metabolic risk for a given age and BMI) would add value to our existing knowledge.

It is clear from prior studies that macrophages represent an important source of Lp-PLA2 [27, 28], and increase with adiposity in omental adipose tissue [29, 30]. Therefore, it was important to ascertain the contribution of macrophages to Lp-PLA2 in adipose tissue, as well as to explore the potential cellular site of Lp-PLA2 expression. In brief, we: (1) evaluated the influence of macrophages, using the macrophage marker EMR1 to explore an association with Lp-PLA2 in adipose tissue [31, 32]; (2) analysed, immunohistochemically, the expression of Lp-PLA2 in adipose tissue; and (3) used isolated mature and differentiated human adipocyte cells to examine Lp-PLA2 and macrophage expression. These studies suggest that adipocytes can be viewed as an important contributing source of Lp-PLA2 expression in adipose tissue and adipocytes. Analysis of the mature adipocytes and adipocyte cell line highlighted that any perceived prior contamination of macrophages in cell cultures did not represent a source of Lp-PLA2 mRNA or protein expression. Therefore, targeting adipocytes in obese individuals with darapladib (an Lp-PLA2 inhibitor known to reduce plaque instability in coronary events [33, 34]) may effectively influence adipose tissue dysfunction in obese/type 2 diabetic individuals with and without CVD.

Prior studies have shown associations between Lp-PLA2 and CVD. Our studies examined circulating Lp-PLA2 and its relationship with selected metabolic markers in type 2 diabetic individuals. The data showed that circulating Lp-PLA2 increased significantly with both the level of adiposity and diabetes status. These findings support previous studies in women with gestational diabetes [33] and South Asian individuals with the metabolic syndrome [34], which report similar increases in Lp-PLA2. Interestingly, relative to people without diabetes, there is a fourfold increase in intracellular Lp-PLA2 levels in adipose tissue from individuals with type 2 diabetes; this may, in part, explain the systemic changes in Lp-PLA2 plasma levels in type 2 diabetic individuals. It should also be noted that other cell types secrete Lp-PLA2, such as monocytes, macrophages, T lymphocytes and mast cells, providing an additional source of circulating Lp-PLA2 in type 2 diabetes [35, 36].

In common with people with CVD, the participants with type 2 diabetes in this study had a more unfavourable cardiometabolic risk profile than the obese individuals without diabetes; this included increased oxLDL and LDL-cholesterol /HDL-cholesterol ratio and diminished HDL-cholesterol levels. Our multivariate stepwise regression analysis confirmed oxLDL, triacylglycerol and HDL-cholesterol as the most important predictors of Lp-PLA2 in participants with type 2 diabetes. Additionally, correlation data showed a clear link between lipid profiles and Lp-PLA2 across different metabolic states. These findings were partially affirmed by a study involving a cohort of men with type 2 diabetes, where a multiple-regression model found triacylglycerols as the key predictor of Lp-PLA2 [37]. Therefore, it appears that an unfavourable circulating lipid profile may drive an increase in Lp-PLA2 in adipose tissue, and this is more pronounced in individuals with type 2 diabetes.

The association of an unfavourable lipid profile with diabetes and the associated increase in intracellular Lp-PLA2 in adipose tissue in type 2 diabetes led us to investigate the importance of the adipocyte in LDL-cholesterol and oxLDL modulation in a human adipocyte cell system. In this cellular system, it was shown that acute oxLDL exposure increased the new synthesis of Lp-PLA2, as observed by increasing gene expression. In comparison, oxLDL treatment in human THP-1 monocyte cell cultures also upregulated Lp-PLA2, suggesting that adipocytes may act in a similar manner to macrophages [21, 38]. Lp-PLA2 protein levels appeared to be induced at an even earlier time point than mRNA expression. This apparent disparity may have arisen because of the capacity of the adipocyte to influx and digest oxLDL and LDL-cholesterol, releasing fatty acid, cholesterol and Lp-PLA2 [39]. As such, Lp-PLA2 is transported into the adipocyte bound to apolipoprotein B on LDL-cholesterol, its primary carrier [40]. Therefore, the protein expression of Lp-PLA2 likely reflects the exogenous Lp-PLA2 available, while the mRNA expression represents the newly synthesised Lp-PLA2. Of note, LDL-cholesterol treatment led to similar findings for Lp-PLA2 protein levels: following acute treatment we observed elevated levels of Lp-PLA2, with a gradual reduction over time. To further implicate Lp-PLA2 in the regulation of lipid mediators, co-treatment of LDL-cholesterol with an Lp-PLA2 inhibitor was tested, resulting in diminished oxLDL-mediated inflammation. Thus, Lp-PLA2 functionality within adipocytes may be required for oxLDL production, and the ability to modify oxLDL production from adipose tissue through the Lp-PLA2 pathway may be an important mechanism to target. Future knockdown studies of Lp-PLA2 could further evaluate these effects on adipocyte cellular functions. This may also ascertain whether adipocyte Lp-PLA2 is a useful therapeutic target to improve the lipid profile of people with type 2 diabetes. While still appreciating that downregulating Lp-PLA2 in adipocytes may be significant in reducing systemic triacylglycerols and oxLDL levels, it should be stressed that, as lipid control is a multifactorial process, examining more than one mediator would enhance our understanding of the potential impact on lipid profiles.

In conclusion, this work has highlighted that AbdSc adipose tissue and the adipocyte may act as separate and significant sources of oxLDL production (i.e. separate from foam cells within unstable atherogenic plaques noted in coronary artery disease) [41]. Furthermore, human adipose tissue and adipocytes appear active sources of Lp-PLA2, with expression induced by LDL-cholesterol and oxLDL. Lp-PLA2 expression is raised in AbdSc adipose tissue from people without diabetes, and this is further enhanced in the type 2 diabetic state. The observed increase of Lp-PLA2 in type 2 diabetic people appears to be associated with an upregulation in systemic lipids. As such, increased Lp-PLA2 protein from adipocytes in obesity and type 2 diabetes may contribute to increased circulating oxLDL levels. In turn, this may further promote inflammation and increase the atherosclerotic risk. Therefore, Lp-PLA2 action within adipocytes appears to represent a novel and important therapeutic target to reduce inflammation, atherosclerotic risk and the development of cardiometabolic complications in type 2 diabetes.

## Electronic supplementary material

ESM Fig. 1 (PDF 133 kb)

## Data Availability

Any data not included within this paper are available from the corresponding author on reasonable request.

## Abbreviations

AbdSc: Abdominal subcutaneous
cPLA2: Calcium-dependent PLA2
CVD: Cardiovascular disease
iPLA2: Calcium-independent PLA2
Lp-PLA2: Lipoprotein-associated PLA2
MAPK: Mitogen-activated protein kinase
oxLDL: Oxidised LDL
PLA2: Phospholipase A2
qPCR: Quantitative PCR
SVF: Stromal vascular fraction
