# Supplementary material for: Differential expression of Lp-PLA2 in obesity and type 2 diabetes and the influence of lipids
Source: Diabetologia. 2018 Feb 9;61(5):1155–66. doi: 10.1007/s00125-018-4558-6 (PMC6449000; doi:10.1007/s00125-018-4558-6)
Supplement: Supplementary file 1 — (PDF 133 kb) [file 125_2018_4558_MOESM1_ESM.pdf]

ESM Fig. 1

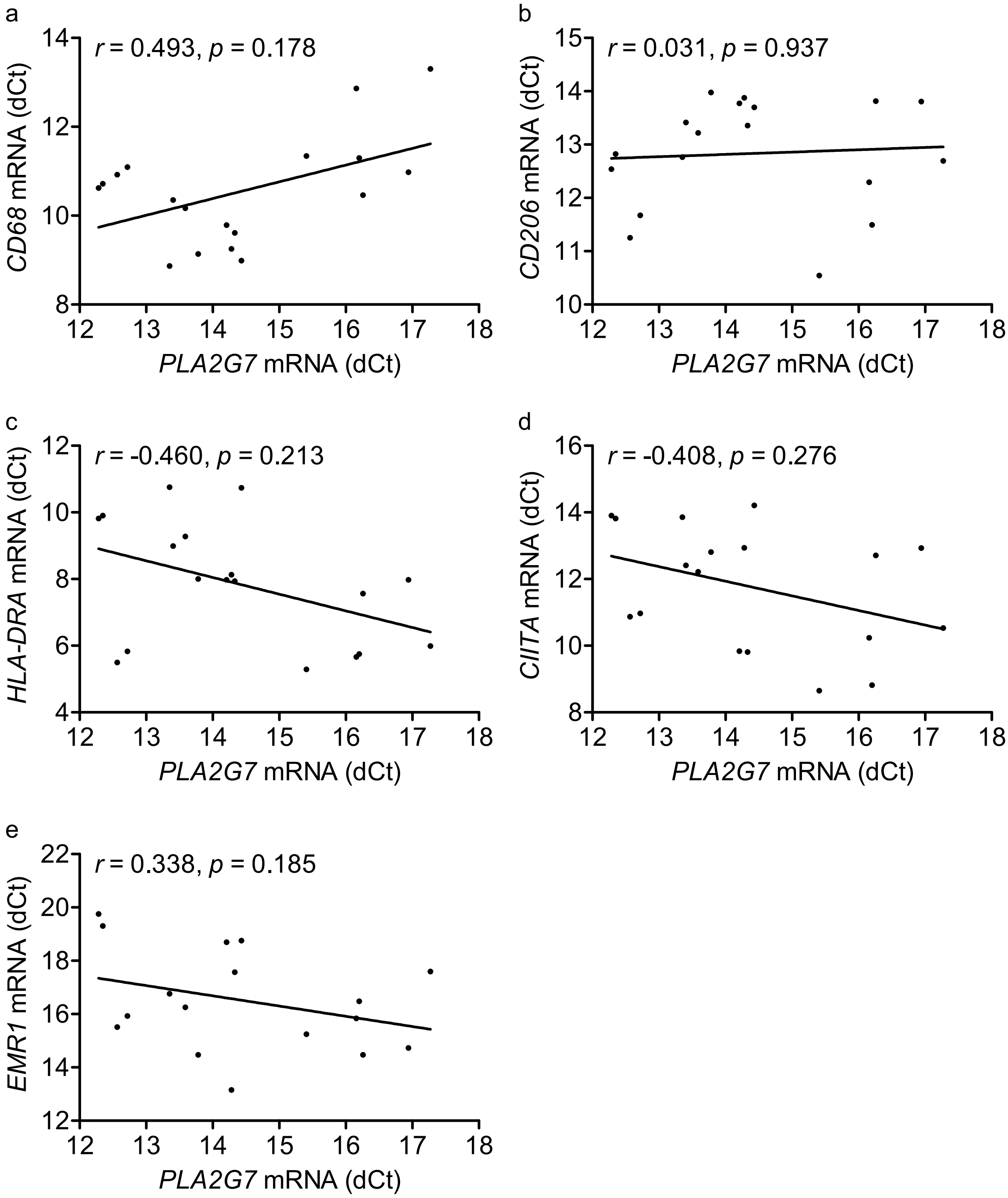

Correlations in SVF and mature adipocytes between *PLA2G7* and **(a)** *CD68*, **(b)** *CD206,* **(c)** *HLA-DRA,* **(d)** *CIITA,* **(e)** *EMR1.* Correlation analysis was performed using Pearson's correlation analysis, followed by two-way ANOVA. $p<0.05$ was considered significant.
